# Abstracts and Gleanings

**Published:** 1879-03-20

**Authors:** 


### ABSTRACTS AND GLEANINGS.

**Pimply-face Acne.**—In a recent lecture by Mr. Jonathan Hutchison, an eminent London surgeon, in which he discusses the whole subject of this unsightly affection, its causes and appearances, he says in regard to the best treatment as follows:

"When the face is covered with pimples, some of which are red, some contain pus, and others show only black points in their centers—all kinds being present, and all show in progress—it is commonly agreed to call the condition acne.

"The rules for the constitutional treatment of acne patients follow easily from what we have said. If the patient be young, he should be made to use a cold bath every morning, to take plenty of exercise, to live liberally as regards meat diet, with a fair allowance of stimulants; and he should be cautioned or encouraged, as the case may be, in reference to sexual matters. As to medicines, a long course of small doses of arsenic will often be of great use. If constipation be present, the habitual use of a chalybeate aperient should be prescribed. You may do all this, however, most assiduously and gain nothing whatever, if you neglect local measures; whilst with the latter only, and without any change in the patient's habits, you may often get an acne eruption so nearly well that he will regard it gratefully as a cure. The chief local measure consists in destroying, by means of a caustic, the inflamed follicles. With a fine-pointed glass brush, or a bit of soft wood cut to a point, you touch the inflamed spots from day to day. Take care not to apply too much. In the left hand should be a roll of blotting-paper with which to absorb the fluid if it has been deposited too abundantly. The best fluid to use is the acid nitrate of mercury. It will usually be necessary to repeat the touching once a week for a month or two, carefully seeking out every fresh spot. After that the patient should still see you once a month, in order that the cure may be kept up. The acid thus used does not leave larger scars than the spots would themselves do.

"In acne rosacea, the use of the caustic will again serve an excellent purpose. You may not only touch the spots themselves, but also pencil out the stray vessels which add so much to the patient's disfigurement. He, or more usually she, will gladly exchange a few slight and scarcely perceptible scars for the angry and very suspicious looking redness of face which the disease causes."—*Med. Times and Gaz.*

**Jaborandi in Puerperal Convulsions.**—Dr. H. B. White relates the following case: Primipara at term, health good until within a few days; strange feeling in her head; swelling of feet and legs coming on for several days and now she could scarcely walk about the room; labor had not begun; had passed but little water for several days—its condition not noted. Infusion of jaborandi was given in repeated doses but convulsions set in with stertorous breathing. But a drenching perspiration soon began, and the patient made a good recovery in the usual time, though the urine showed moderate amount of albumen.

**Rules for Sending Consumptives to Travel.**—The following rules are those laid down by Dr. James Edward Pollock, in a recent lecture:

1. Never permit any patient to travel who is not in the quiescent stage of disease, or who, in other words, is feverish, with high evening temperature, and the physical signs and conditions already described to you, indicating the continuous form of phthisis. Observe this rule, and you will be successful; break it and your patient and his friends will not thank you.

2. None of the secondary complications should be present, as continuous or frequent diarrhœa, serious gastric disorder, or laryngeal irritation.

3. Chronic single cavity, with retraction of walls, accomplished or proceeding, is favorable for removal to a dry, bracing locality, if the hæmoptysical element be wanting in the case.

4. That form of disease described as diffused deposit in one lung, without much dullness or signs of massing of disease, with pretty large chest, and with more moderate emaciation, generally does well on a sea voyage.

5. A first-stage case, already chronic, does best for traveling about, with frequent change of residence. The complication with bronchitis or asthma is generally much benefitted by change.

6. Persons ought not to travel *at all* with feverish symptoms; with secondary complications; with a large amount of local disease in any stage; with both lungs diseased, with poor digestion and greatly lowered nutrition; or in such a state of weakness or emaciation as to require home comforts, peculiar beds or chairs, or varieties of invalid cookery.—*Medical and Surgical Reporter.*

**Phimosis as a cause of Rupture in Children.**—Mr. J. Arthur Kempe reports, in the Lancet, a series of observations which leads him to believe that phimosis is not an unfrequent cause of hernia in infants. He states that the sequel of events is probably as follows: The abdominal parietes are naturally weak in children, which renders them less able to resist impulses which project the viscera against weakened parts. Here, then, is a remote or predisposing cause. The exciting cause is readily supplied by the frequent and continued efforts that the child makes to overcome the obstruction offered by the tight prepuce, and by the cries uttered consequent on pain caused in making these efforts.—*Ibid.*

**Anorexia.**—Dr. Fonssagrives recommends the following formula: Bruised Chinese rhubarb, bruised peel of bitter orange, of each four parts, and water 250 parts. This is to be infused cold during three days. Two or three tablespoonfuls are to be taken daily an hour before dinner. If this does not succeed, he prescribes one or two pills an hour before dinner, each containing one centigramme of alcoholic extract ol nux vomica, and twenty centigrammes of extract of gentian, continuing them until the appetite is suitably restored.—*Ibid.*

**Milk Diet in Cystitis.**—The Lancet reports a case of chronic cystitis, occurring seven years after lithotrity, as having been cured by an exclusive milk diet.

**Notes from Practice.**—I have jotted down some points of experience which I think are worth preserving. Let your readers take them at their value :

1. Hydrobromic acid is a good brain and nerve sedative in acute melancholia (dose ʒj to syr. simp. ℥ss), but after a week's use disorders digestion. It is too disagreeable to administer to females.

2. Tannic acid, in powdered form, applied to wounds constituting compound fractures, will convert them, when the wounds are not extensive or torn, into simple fractures, by rapidly forming a cicatrix, and thus save from one-third to one-half the usual time of healing.

3. An ounce or two of oil of turpentine in half a pint or a pint of tepid menstruum, arrests, like a charm, intestinal hemorrhage, even when patients seem moribund.

4. There is a form of bilious puerperal fever in which, by means of severe diarrhœa, nature effects a cure. To arrest this is bad practice and uniformly fatal.

5. I have reduced gradually to normal frequency, and thus saved the patient, a pulse in puerperal fever that ran 150 beats per minute, by half-drop doses Norwood's tincture veratrum viride, with one grain quinine, administered every two, three and four hours.

6. I recently examined several patients in Brigham Hall Asylum, by "cranial percussion," with a view to "cerebral localization." In a respectable percentage I succeeded. In one epileptic lady, whose skull was broken many years ago, I was enabled to point out distinctly the seat of fracture.

7. In summer diarrhœa of children (attention to all other hygienic conditions understood) the following treatment has almost worked miracles in my hands :

℞ Calomel........................... gr. j<br>

Soda bicarb......................... grs. vj<br>

Ft. chart No vi.

SIG.—One every hour until used, then one teaspoonful of castor oil, after which :

℞ Extract hæmatoxyli..................... gr. j<br>

Tr. kino................................ ʒ ss<br>

Syr. simplicis..........................<br>

Aq. menthæ pip.........................aa ℥j M.

SIG.—According to age ʒss to ʒj, twice daily.

8. Pleasant, odorous substances are of greater value, curative and preventive, than the faculty accredit them.

A form of administering iron, more natural, convenient and efficacious than hypodermically, a form in which it will be borne, liked and appropriated by every patient in whom it is indicated, from the infant to the aged, is as follows :

℞ Oil of wintergreen........................ gtts. xvj<br>

Alcohol..................................... ℥x<br>

Whisky...................................... ʒ xij<br>

Shake and add<br>

Water....................................... ℥ xxxij<br>

Sugar....................................... ℔j<br>

Fl. ext. gentian............................ ʒj<br>

Iron, pyrophosp. (or) am. cit............... ʒj¼ Shake well.

SIG.—A teaspoonful twice daily, before meals, in ʒss water.—Dr. R. J. Hutton, in *Med. and Surg. Peporter.*

**Prognosis in Cerebral Hemorrhage.**—It is often important to be able to give a reasonably correct opinion as to the result of apoplectic attacks, in answer to inquiries by friends and parties interested. Dr. Lapponi, in the Revista Clinica de Bologna, presents some valuable hints on this subject, whichmay be epitomized as follows :

Those attacks in which coma continues over twenty-four hours are fatal. There are a few exceptions which extend the farthest limit to three days.

There are but few attacks followed by slightly prolonged coma, in which one fails to observe, before the return of consciousness, occasional yawnings separated by intervals more or less prolonged. But if these yawnings occur soon after the attack, if they are frequent and succeed each other rapidly, a fatal termination is certain.

Paralysis of the buccinator always indicates a serious attack, as the seat of lesion is not far from the medulla oblongata. Equally grave, and perhaps more so, is labio-glosso laryngeal paralysis, which the author thinks he was the first to observe. Here the paralysis is of the hypoglossal and a portion of the facial nerve, from lesion of bulb.

All cases in which, thirty or forty minutes after the attack, vomiting occurs without nausea or effort, being a veritable regurgitation of the stomach, will terminate in death. The value of this symptom is due to lesion of the vagus nerve.

Paralysis of the pharynx, from lesion of the origin of the vagus, and polyuria supervening a few hours after the attack and due to lesion of the bulb, alike indicate great danger.

Extreme depression of temperature occurring soon after the attack, is often the prelude of death. But if there succeed to this initial fall of temperature a reaction which raises the temperature above the normal standard, the prognosis is unfavorable without exception.

Finally, the decubitus acutus, so well described by Charcot, is a fatal symptom.—*Pacific Med. Journal.*

**Treatment of Enuresis Nocturna.**—Dr. Kelp has, on several occasions, drawn attention to the value of subcutaneous injections of the nitrate of strychnia in the treatment of obstinate cases of nocturnal incontinence. He practices the injections in the neighborhood of the sacrum. A single injection of a very small quantity of the drug suffices o arrest the affection for a certain time, and when it reappears, the operation can be repeated. His later paper cites the case of a young woman, eighteen years of age, who had suffered from enuresis every night for several months; it came on first after an attack of scarlatina, and persisted in spite of all precautions. The first injection procured her a respite of several nights, and the second produced a permanent cure. The patient was a strong, healthy girl, and had never suffered from enuresis previous to the attack from scarlatina.—*Le Mouvement Medical.*

**Oil of Aleurites Triloba as a Cathartic.**—Dr. Oxamendi esteems the oil obtained from the aleurites triloba, known in Ceylon under the name of kekune oil, as the best substitute for castor oil. It has a pleasant taste like that of hazelnut oil, and causes no unpleasant effects. Half an ounce is the dose for an adult; it operates in about three hours without causing pain or colic.—*Allg. Med. Cent. Zeit.*

**Picrate of Ammonia in Hooping-cough and Diphtheria.** Dr. Z. T. Dellenbaugh, of Philadelphia, has been experimenting with picrate of ammonia, and in the Philadelphia Medical Times, August 31st, 1878, says that since a former article on that subject (May 25th) in that journal, he has had ten or twelve additional cases of hooping-cough, which he has treated with picrate of ammonia with the most satisfactory results. "Indeed, some of the cases were cured in the marvelously short time of from twenty-four to seventy-two hours." In view of this experience, together with some fifteen or twenty cases reported to him within the past few days, Dr. Dellenbaugh thinks he can most safely affirm that, if properly administered, the picrate of ammonia is a specific for the cure of hooping-cough. He gives to babies from one-sixteenth to one-twelfth grain, and to children from one-twelfth to one-eighth grain, every three hours. In one case of diphtheria, he has also used picrate of ammonia as a gargle (gr. viij to Oj), and by atomization. The solution of picrate produced a yellowish staining of the parts in such a way that he was inclined to believe a destruction of the micrococci ensued, and a speedy cure of the disease was the result. It, of eourse, will be advisable to detach thick exudations so that the picrate of ammonia solution can come in direct contact with the colonies of spherical bacteria.

**Benzoate of Lithia.**—Dr. T. O. Summers says: "In our hands benzoate of lithia has often a most magical effect in diminishing the uric acid deposits and increasing the free hippuric acid of the urine. It will thus be seen that this agent acts in a manner entirely different from the alkalies in the cure of uric acid deposits. Benzoate of lithia acts upon the urine before it leaves the blood, converting the uric acid, which would otherwise be deposited, into hippuric acid, a harmless agent, which passes off in solution, leaving no ill effects. The alkalies do not act in this way. They dissolve the uric acid directly after it has left the blood. Hence, while they are clearly indicated in those cases in which the acidity of the urine is so great as to produce irritability of the bladder or any part of the genito-urinary apparatus, it is clear that they cannot change the lithic acid diathesis—an end which we may hope to attain according to the rationale of the benzoate of lithia action." —*Nashville Journal of Med. and Surg.*

**How to Cough.**—To some persons coughing is harmless, but to others it is fraught with many dangers. It is, therefore, important to teach those liable to be injured by too severe or prolonged efforts at coughing how they may accomplish their purpose easily, safely and quickly. Dr. J. M. Fothergill (Philadelphia Medical Times) says: "It must be insisted upon that the chest be well filled with air before the cough is let loose; that is, the reflex act must be inhibited by the exercise of the will, until the chest be well filled with air before the cough is let loose. Such full inspiration is effective not only in removing the source of the irritation, but it usually causes other masses of mucus and charcoal to slide from their seat, and thus to set up further cough for their removal. But if the full inspiration plan be followed, these masses are readily and quickly expelled." Of course, these directions are of use only in such coughs as are for the purpose of removing some offending matter from the air passage.—*Cin. Lancet and Clinic.*

**Carbolic Acid Spray in Coughs.**—A correspondent, who is a druggist in this city, sends us the following communication on this subject:

More than a year ago I read in the Journal of Chemistry a statement that carbolic acid in the form of spray benefitted a cough. Having a severe cough at that time, I used the acid as directed, of a strength of about two per cent., with an atomizer, but finally tried five per cent., or the saturated solution. I took no medicine, and the cough went away in a few days. Now, from my first recollection I have had severe coughs, and have always had bronchitis, for which I have taken much medicine; but since using the carbolic spray I have had no cough for a year. If I feel any of the symptoms which precede a cough or a cold, a few inhalations remove all the disagreeable feelings, and prevent a cough. Inhalation through the nostrils stops sneezing and the flow of mucus. I have recommended it to many others, all of whom were benefitted, and cured if they contiuue to inhale the spray.

I have called the attention of many physicians to the value of carbolic acid in coughs, asthma, and chronic catarrh, and to the fact that the saturated solution (five per cent.) could be used with safety, and would in most cases be more beneficial than a weaker solution. They have answered that they would not give the acid of that strength under any consideration. But I have often used it of that strength, and many other people have tried it, with no other effect than soothing the irritation of the membrane to which the spray was applied. The tickling sensation soon ceases, and the mucus is raised with but little effort. In fact, it relieves all the unpleasant symptoms and stops the progress of the catarrh. I believe that it is an absolute cure for all inflammations of the mucus membranes of the nose, throat and lungs, and that it produces the desired effect immediately by contact with the affected part.—*Boston Journal of Medicine.*

**Hypodermic Injection in Hernia.**—Reporting upon three cases communicated to the Societe de Chirurgie, in which strangulated inguinal hernia was easily reduced after the hypodermic injection of morphia, M. Le Dentu observes that in these cases the strangulation was recent, and although the injections certainly assisted their reduction, it is doubtful how far they would have succeeded had the strangulation been more decided and of longer duration. If the surgeon is called to the case immediately, the injection may be of use by dissipating the pain and spasms; but if some hours have elapsed, it will be always of less value than chloroform, which enables us to at once recognize whether the hernia is reducible or the operation necessary.—*Gaz. des Hopitaux.*

**Treatment of Baldness.**—The following is highly commented upon by Dr. George H. Rowe, for seborrhœa and consequent alopecia. It is the plan of Prof. Kaposi. ℞. Saponis viridis (German), alcoholis, aa. f. ℥ ij.; solve, filtra, et adde ol. lavandulæ, gtt. xx.—xxx. Pour one or two tablespoonfuls upon the scalp, then pour on a little water, rub smartly with the fingers, thus producing a copious lather. After four or five minutes' shampooing this way, rinse the head thoroughly with pure water, then dry thoroughly with a towel. Then apply a little cosmoline. This process causes the hair to fall out in greater abundance at first, but a new and fine growth of hair soon follows.

**On the Treatment of Morbus Coxarius by a New Method.**—Dr. Joseph C. Hutchinson, in an article (Amer. Journal of Med. Science), advocates the following plan of carrying out the indications for the treatment of morbus coxarius, which he considers to be: 1st. To secure immobility of the joint; 2d, to procure extension of the limb; 3d, to take off from it the superincumbent weight of the body; 4th, to provide means to enable the patient to take exercise in the open air. He considers that immobility is obtained by the rigidity of the joint, and that this continues until nature says it is no longer necessary. To obtain extension of the limb and to remove the weight of the body he resorts to the following device: On the shoe of the sound limb an iron sole is applied, three inches high, so as to raise the foot from the ground. This elevated shoe and a pair of crutches constitute the apparatus. As the patient stands on his crutches the diseased limb is suspended. The shoe should be high enough to prevent the toes of the affected limb from touching the ground.

By these simple means we fulfil all the indications for the mechanical treatment of hip-joint disease. Immobility is obtained and friction prevented in the manner above indicatcd—chiefly by rigidity of the periarticular muscles. Extension made by the weight of the suspended limbs, which is greater than the weight ordinarily employed for extension, is quite sufficient to relieve the inflamed parts from pressure and pain, and to overcome deformity of the limb even though it be considerable; the weight of the body is removed from the diseased joint, and the patient can enjoy all the benefits of open-air exercise.—*Med. Rec.*

**The Treatment of Tape-worm.**—Prof. Mosler has been advocating a system of treating tape-worm which, according to a Swiss medical journal, has been attended with remarkable success. Its chief characteristic is the injection of large quantities of warm water into the colon after the administration of the anthelmintic. The diet is first regulated, food being given which is supposed to be distasteful to the tape-worm—bilberry tea, sour cucumber, salted meats. The intestine having been, as far as possible, emptied by laxatives, a dose of the extract of pomegranate bark is administered, prepared from the fresh bark, and then a large quantity of warm water is injected into the rectum. The theory is that the worm, previously brought down into the colon, is prevented by the water from attaching itself to the wall, and is brought away by the liquid on its escape. It is asserted that in every case in which this treatment was adopted the head of the worm was removed.—*London Lancet.*

**Treatment of Cerebral Apoplexy by Injections of Ergotine.**—Mr. Foster reports two cases of pronounced apoplectic coma, which were treated by the injection of twelve drops of a solution of ergotine, containing seven and a half grains to the drachm of vehicle. In both cases the coma disappeared soon, and recovery took place. The essential condition for the success of the treatment is, that the injection be administered at the beginning of the attack, before there has been time for an extensive extravasation of blood. Mr. Foster recommends injecting the fluid between the muscles of the forearm, and not merely under the skin, where it is liable to excite suppuration.—*The Lancet.*

**How to Give Electric Baths.**—A correspondent of an English contemporary writes :

"There are several ways of making or giving the electric or galvanic bath. One is, having a wooden bath lined with copper, and having a crossbar of wood and copper for the patient to hold as he lies in the water. One of the cords from the battery is attached to either the bar or the copper part of the bath, which becomes the positive pole ; the negative is placed in the water. The water should be from 85° to 95°. Another method, which is much easier of application, is used by me constantly. It is an ordinary long dressing-room bath, two parts filled with water, 90°, so that the patient can lie down comfortably ; the water is then charged by placing two plates of copper (size 12 by 6) into the water ; the plates are attached to the cords from a Meyer and Meltzer continuous current battery. The plates must not be allowed to touch the person, for a sharp burning is at once felt, and they will almost jump out of the bath, from the sudden pain. I have seen a patient after twenty minutes in a bath of this kind look just as a boiled lobster, the skin being very red, and giving a tingling sensation for some little time. The diseases for which I am in the habit of using it chiefly are plumbism, lead colic and dropped wrist, rheumatism (subacute and chronic), nervous debility, amenorrhœa, want of development of the uterine organs, infantile paralysis, paralysis agitans, progressive locomotor ataxy, and impotency ; and considering that nearly all these diseases are of a slow or chronic type, this kind of treatment has been most successful."—*Med. and Surg. Rep.*

**Sulphc-Thymate of Quinia.**—Signor Cozzolino recommends the *sulpho-thymate of quinia*—a compound of sulphuric and thymic (thymol) acids and quinia—as worthy of ranking beside the sulpho-carbolate or salicycate of the same alkaloid. It is freely soluble in acidulated water, in ether, and in alcohol. Dose is the same as the above-mentioned salts. He also calls attention to soda-thymate as a pleasant carminative (dose : 0.50 gmm. for infants ; 3.00 to 4.00 gmm. for adults) mouth-wash in aphtha and muquet, as an injection in vaginal, uterine and vesical affections.

**Treatment of Distemper.**—It will be interesting to lovers of the canine species to hear of a simple remedy for distemper. At the quarterly meeting of the Scottish Metropolitan Veterinary Medical Scc'ety Mr. Baird mentioned the case of a collie dog in the last stage of the disease, and which its owner had determined to destroy. Shortly after being treated with doses of strong coffee and a little sweet milk, the animal, however, so far recovered as to be able to stand and walk. The chairman of the meeting said the case seemed almost unique.—*London Lancet.*

**Onychia Scrofulosa.**—In the treatment of onychia scrofulosa, which is always accompanied by fungosities, M. de Saint-Germain employs very simple operative procedure. It consists simply in paring off the end of the finger or toe, as one would trim a quill-pen, removing in one sweep the nail and its matrix, and the superficial portion of the phalanx with the fungosities developed on it. The resulting wound heals very rapidly.—*Journal de Medecine et de Chirurgie.*

**Anteflexion of the Womb.**—The treatment of this disease at the Pennsylvania Hospital is peculiar. No pessary and no mechanical instrument of any kind is employed. Dr. John Forsyth Meigs does not believe at all in the use of pessaries in anteflexion. For the patient's general health, f. ℥ ss. of the compound gentian mixture is ordered to be taken thrice daily with gr. iv. of the ammonio-citrate of iron. To clear out the bowels and keep them open the following mixture is prescribed:

| | | |
|---|---|---|
| ℞. Magnesii sulphat | ʒ vj. | |
| Acid. sulph. dil | ʒ ij. | |
| Ferri sulphat | gr. xij. | |
| Quiniæ sulphat | gr. xij. | |
| Syrupi zingiberis | f. ℥ j. | |
| Aquæ q. s. ad. | f. ℥ vj. | M. |

S. A tablespoonful in ice-water three times a day.

This not only acts as a gentle and constant aperient, but also invigorates the appetite and promotes the general health.

To remedy the condition of anteflexion, the woman's bladder is taught to hold each day a larger and larger quantity of urine—from f. ℥ ij–iv–vj–viij–x., until it finally can retain f. ℥ xij. By that time the anteflexion is largely reduced. The fuller the bladder is, the more rigidly does the uterus regain its normal position.—*Hospital Gazette.*

**A New, Cheap and Self-generating Disinfectant.**—Under this title, Dr. John Day, of Geelong, Australia, recommends for use in civil and military hospitals, and also for the purpose of destroying the poison-germs of small-pox, scarlet fever, and other infectious diseases, a disinfectant ingeniously composed of one part of rectified oil of turpentine and seven parts of benzine, with the addition of five drops of oil of verbena to each ounce. Its purifying and disinfecting properties are due to the power which is possessed by each of its ingredients of absorbing atmospheric oxygen and converting it into peroxide of hydrogen—a highly active oxidizing agent, and very similar in its nature to ozone.

**Treatment of Albuminuria by Fuchsine and Rosaniline.**—Feltz and Bouchut have treated a small number of patients suffering from albuminaria with fuchsine and rosaniline, given in pills and mixture, in doses of three grains per diem. Under this treatment the albumen soon disappeared from the urine. They claim that these therapeutique trials prove at all events that both these coloring agents are well borne by the organism, and are relatively harmless.—*Deutsche Med. Wochen.*

**Cracked Nipples.**—Dr. Haussman has found that lint, soaked in a two per cent. solution of carbolic acid, applied to the nipples, and wetted every two or three hours with the same, gives immediate relief to the pain, and causes complete healing (although the baby is still nursed from the nipples) in two or three days.—*Ib.*

**Insufflation Powders *vs.* Nasal Douche.**—Dr. H. G. Miller, of Providence, deprecates the use of the nasal douche, and insists all medications should be in the form of dry powder and used by insufflation.—*Proc. R. I. Medical Society.*

**Viburnum Prunifolium for Controlling the Habit to Abortion.**—Dr. Jenks gives a very interesting and practical outline of his experience in the use of this article. He states that where the habit of aborting has been formed, his mode for prescribing the viburnum is to have the patient take from half a teaspoonful to a teaspoonful of the fluid extract four times a day, beginning at least two days before the regular menstrual date, and continuing it, not only during the period of the catamenial flow, but two days longer than that discharge continues when the woman is not pregnant. If special urgency exist, teaspoonful doses may be given every two or three hours. He designates viburnum prunifolium as a uterine sedative whose action is as pronounced as is that of ergot in producing uterine contraction. It is not alone in the prevention of abortion that it proves by virtue of this peculiar sedative action a most valuable therapeutic agent; it proves equally efficient in the treatment of the sympathetic disoiders incident to pregnancy, where a nervine or sedative is demanded, and in a large class of non-puerperal diseases of women. The use of viburnum in this last mentioned class of cases deserves more attention than it has hitherto received.—*Obstetric Gazette.*

**Liquor Bismuthi for Nasal Catarrh.**—Dr. Q. C. Smith writes to the Pacific *Medical Journal*, recommending for nasal catarrh liquor bismuthi and water,, equal parts, applied one to three times a day to nostrils, pharynx and naso-pharyngeal cavity, freely, with a spray producer. He has found this during an experience of several months, to produce very satisfactory results. Sulpho-carbolate of zinc, in week solution, as mentioned by other writers, he regards also as a very efficient remedy; applied in the same manner.—*Med. Press.*

**Nitrite of Amyl in Infantile Convulsions.**—In a case reported by Dr. Engel, this agent was successfully used. The parents had lost three children previously by epileptiform convulsions of the same character as those affecting the present case. The child, eighteen months old, has continued in convulsions for five hours, and was apparently moribund, when as a last resort five drops of the amyl were given along with ¼ gr. of morphia. The child at once went off into a quiet sleep. —*Phil. Medical Times.*

**Post-Partum Hemorrhage.**—Dr. Penrose used vinegar in the following manner : Saturate a rag with vinegar, carry it into the cavity of the uterus, and squeeze it. In the vast majority of cases the hemorrhage ceased as by magic when the vinegar passed over the surface of the uterus and the vagina. He believed that the salts of iron should seldom or never be used, but in a case, which was supposable, in which the vinegar failed he would resort to that remedy.—*Ex.*

**Injecting a Tumor with Morphia before Extirpation.**—Half a grain of sulph. morph. with a thirty-sixth of a grain of sulph. atoipiæ, was injected in a fibrous tumor on the upper arm weighing about a pound, and its removal accomplished without pain. The case was reported to the North Carolina Medical Society by Dr. Foote, its late president. A second case was mentioned with the same result. In both instances sleep came on only after several hours.—*Ex.*

**Simple Treatment of Quinsy.**—Leslie Thain (Canadian Medical Journal) believes gargles of alum, tannic acid, and such similar astringents, useless for the purpose of astringing the vessels sufficiently to "press back" the inflammation. His plan is to apply externally hot fomentations (with a few drops of turpentine) to the throat, and then to wrap up the whole neck in flannel. Constant heat, moisture and mild counter-irritation are to be kept up by frequent changing of these applications. The feet must at once be placed in a hot mustard bath, and if the patient will then get into bed between two blankets, so much the better.

Gargles as hot as can be borne must be begun as soon as possible, and the most useful is a solution of carbolic acid, one part to forty of water. If the patient cannot gargle, carbolic acid in glycerin (one to twenty or thirty) should be frequently applied by means of a feather to the parts. A brisk saline aperient may be advisable. By following this plan of treatment, the inflammation subsides in a few hours, never running on to suppuration, and then a simple alum gargle may be serviceable.—*Phil. Med. Times.*

**Treatment of Gleet.**—Of late months I have undergone a change of opinion in the matter of the treatment of gleet. I now think that there is nothing in the world so good as the introduction of nickel-plated conical bougies and the simple overstretching of the inflamed parts.

Before proceeding to use an instrument of this sort, however, it is necessary in all cases to measure the normal calibre of the rest of the urethra, so that you may be guided in the selection of a bougie of the proper size.—S. W. GROSS, M.D., in *N. Y. Medical Record.*

**Neuroses of the Heart.**—J. Milner Forthergill, discussing the neuroses of the heart, divides the anginose affections into neurosa angina and true angina pectoris. The former is oftenest seen in women at about the climacteric. It is not very dangerous, and is frequently relieved by arsenic. True angina is produced by spasm of the arterioles, which causes a rise of pressure within the heart. It occurs oftenest in men of gouty condition and may be relieved sometimes by iodide of potash.—*Brain.*

**Graafian Vesicle during Pregnancy.**—Dr. Slaviansky reports (Med. Centralzeitung) the case of a woman, æt. 24 years, who died in the third month of gestation, and the post-mortem showed ovarian follicles which were on the point of bursting, as well as recent *corpora lutea.* This confirms the opinion enunciated by the late Prof. Charles D. Meigs, that the development of the Graafian follicles continued during pregnancy.

**To prevent Boils.**—A very simple remedy is made known by Dr. Sieven, in a St. Petersburg journal, for preventing the development of boils. He states that if the skin be superficially scraped with a small knife, so that a drop or two of blood may be pressed through the epidermis as soon as the peculiar stabbing or pricking sensation and slight induration announce the commencement of the boil, it will not be further developed.

**Dry Dressings in Wounds.**—Mr. Sampson Gamgee, in the Lancet, advocates the treatment of wounds by dry and infrequent dressings, uniform pressure and absolute rest. The following is his plan: He unites the edges of the wounds with silver sutures; a gauze and oakum pad is then placed over the wound, the limb enveloped in cotton-wool sufficiently to protect the bony prominences, and immobilized by lateral and posterior moistened pasteboard splints from foot to hip. A gently-compressing bandage completes the dressing. He gives the histories of three cases of injury about the knee-joint, treated on this plan, and all did well. In one the tendon of the quadriceps extensor was completely divided, the intercondyloid space exposed, and the finger could be passed underneath the patella. The wound was not laid bare until the ninth day; healing was then perfect. There had been neither pain nor rise of temperature. A case of extensive contused and lacerated wound of head was also successfully treated on this plan. —*Ibid.*

**Vulcanized India-Rubber in Blepharitis.**—The treatment consists in the application every evening to the affected eye of a round plate of caoutchouc, which is covered with a compressive bandage. In the morning the apparatus is removed, and the eye washed with warm water. Dr. Roy reports several cases treated in this way, with very gratifying success. The only phenomenon noticed, on removing the apparatus in the mornings, was a slight redness of the eye.—*Lyons Medical.*

**Tincture of Myrrh in Whooping-cough.**—According to Dr. Campardon, pertussis yields readily and easily to the tincture of myrrh. He gives fifteen drops in a tablespoonful of Vichy-water every hour or every two hours. This treatment, however, must be combined with the appropriate treatment for the bronchitis or the pulmonary congestion.—*Ibid.*

**Pilocarpin in Albuminuria.**—Dr. T. G. Thomas regards it as a serious error when a physician thought it necessary to produce premature labor in every case of albuminuria. In the treatment of these cases he regards it as important to keep up the excessive action of the skin, and for that purpose pilocarpin was suggested as a most reliable diaphoretic.

**Obstetric Forceps.**—Dr. A. H. Smith, of Philadelphia, takes the position that, for the safety of the mother and the child, the obstetric forceps should be kept in the median line and should not receive any lateral or pendulum motion. Dr. Smith regards the leverage power of the forceps as a most dangerous delusion, and that as an aid to the traction efforts of the forceps it had no foundation in fact.—*Obst. Gazette.*

**Sulphurous Acid in Scarlet Fever.**—Dr. L. Waterman, of Indianapolis, reports his experience in an epidemic during the year 1876. He says: "I early adopted, on antizymotic principles, the administration of from ten to thirty drops every two, three or four hours, of sulphurous acid diluted. I treated eleven severe cases. The ten treated after its adoption recovered."—*Ex.*

**Acetate of Potass. in Pneumonia and Rheumatic Fever.**—Dr. T. O. Summers says: "In pneumonia and rheumatic fever the quantity of free acid is much greater than in health. In both these diseases we have seen a more ready yielding to the alkaline than to any other. We have administered as much as one ounce of acetate of potash during twenty-four hours with almost magical effect. In fact in almost all inflammatory diseases great relief is experienced by this line of treatment. The whofe direction of osmosis is changed, and the exudations of inflammation are altered by it more sensibly than by any of the much vaunted antiphlogistic measures so generally adopted."—*Nashville Journal of Med. and Surg.*

**Treatment of Uric Acid Diathesis.**—Dr. T. O. Summers, after advising the saturation of the urine by the alkaline treatment for months, remarks: "The treatment of the diathesis, in the meantime, is generally directed to the digestive system; and here we would direct especial attention to the valuable therapeutic effect of pepsia, combined with phosphate of ammonia. They should be combined in the proportion of fifteen grains each and taken at each meal, mixed with grits, hominy or rice. The phosphate of ammonia has an agreeable flavor and may be used instead of salt."—*Ibid.*

**Wounds.**—Dr. G. F. Waters, of Boston, has found the milky juice of the common milk-weed (*Asclepias Syriaca*) to afford an elegant method of healing wounds. The only essential point is to dry the wounded surface gently and thoroughly with blotting-paper before applying the milk-weed juice. From the description, it appears that after the juice was applied, and while the healing was in progress, a piece of blotting-paper was also used to cover the surface.—*Exchange.*

**Danger of Opium in Chronic Bright's Disease.**—The possibly dangerous effect of even small doses of morphia in chronic Bright's disease, although alluded to by several writers of authority, is not so widely and thoroughly known as it should be by the profession. Intolerance of this drug is one of the peculiarities of the disease: doses so small as to be looked upon as safe under any circumstances will sometimes have a poisonous effect.—*Phil. Med. Times.*

**Another Anti-emetic.**—The value of spiritus nucis juglandis, "spirit of walnut" in vomiting, is much insisted on by Dr. E. Mackey. He has used it in various forms of vomiting, and found it successful after other things failed. He gives it in drachm doses three times a day.—*Practitioner*

**Colliquative Diarrhœa.**—Dr. Bonfigli recommends most highly chlorate of potassium in colliquative or nervous diarrhœa without intestinal lesion. He advises the increase of the dose until a beneficial effect is observed, beginning at half a drachm in the twenty-four hours, and increasing to two or three drachms if requisite.

**Oleate of Zinc.**—Either as an ointment or in solution, oleate of zinc is highly recommended by Dr. Crocker, of London, in eczema, chronic ulcers, etc.

**A New and Successful Treatment of Shock.**—Dr. Charles T. Hunter, Demonstrator of Surgery in the Medical School of the University of Pennsylvania, has lately introduced a new and successful treatment for the general shock following railroad injuries, etc. The patient is at once placed in a bath of 98° F.; the temperature of the bath is then rapidly raised to 110° F. As is well known, the temperature of patients suffering from shock is as low as 96° in the armpit. By this method of treatment, Dr. Hunter has been able to raise the patient's temperature from 96° to 98½, and to reduce his respirations in number from 36 to 20 in the minute. Before the bath, the skin is cold and clammy; on taking the patient out, it is warm and dry. The patient is kept in the bath from ten to fifteen minutes. This treatment has been followed in a number of recent cases in the surgical wards of the University Hospital.—*N. Y. Med. Rec.*

**Ice in the Rectum in Chloroform Narcosis.**—According to Dr. Baillee, there is no more active remedy in the narcosis caused by chloroform than the introduction of a piece of ice into the rectum. A moderate pressure suffices to overcome the resistance of the sphincter. The ice melts in the rectum, and immediately a deep inspiration is produced, the precursor of natural respiration and of the re-establishment of the cardiac functions. Dr. Baillee recommends the same means in apparent death of the newborn.—*Gaz. des Hopitaux.*

**Bryonia and Drosera in Whooping-Cough.**—During the catarrhal stage, give the tincture of bryonia (f ʒ ss, daily to a child aged seven years). During the spasmodic stage, give tincture of drosera (same dose for equal age). Under the action of the latter, the disease very rapidly subsides. So says Mr. Louvet Lamare in *L'Annee Medicale.*

**Icteric Urine.**—Jaundice may be diagnosticated by an examination of the urine with tincture iodine. Pour a little of the tincture upon the urine, do not shake the tube, and three distinct colored layers may be seen—first layer, violet, the tincture itself; below this, a sea-green layer; and the last, the urine in its original color.—*Medical Record.*

**Milk Diet.**—In the London Lancet for December 16th is a clinical lecture by Dr. Geo. Johnson on the use of milk diet, which he commends most highly in chronic diarrhœa, dysentery and acute Bright's disease. The chief stress is, however, laid upon the value of the method in acute and chronic cystitis, and one case of rapid and complete cure in a very severe case of two years' duration is reported.

**Tetanus and Chloroform.**—From an analysis of 415 cases of tetanus, Dr. D. W. Yandel asserts that chloroform is the most efficient agent in its treatment, while calabar bean ranks among the least effective.—*Brain.*

**Phosphide of Zinc,** in a granule of from one to two-fifteenths of a grain, thrice daily, seems to have proved an effectual remedy for hysteria, in the hands of Dr. Gross.—*Canada Med. Rec.*

